# Targeting Disulfidptosis with Potentially Bioactive Natural Products in Metabolic Cancer Therapy

**DOI:** 10.3390/metabo14110604

**Published:** 2024-11-08

**Authors:** Xinyan Li, Jiayi Xu, Liangwen Yan, Shenkang Tang, Yinggang Zhang, Mengjiao Shi, Pengfei Liu

**Affiliations:** 1 Department of General Surgery, National & Local Joint Engineering Research Center of Biodiagnosis and Biotherapy, The Second Affiliated Hospital of Xi’an Jiaotong University, Xi’an 710004, China; lixinyan1205@stu.xjtu.edu.cn; 2 International Joint Research Center on Cell Stress and Disease Diagnosis and Therapy, National & Local Joint Engineering Research Center of Biodiagnosis and Biotherapy, The Second Affiliated Hospital of Xi’an Jiaotong University, Xi’an 710004, China; xujiayisprint@stu.xjtu.edu.cn (J.X.); yanliangwen@stu.xjtu.edu.cn (L.Y.); 222020921420@email.sntcm.edu.cn (S.T.); zyg970726@xjtu.edu.cn (Y.Z.); 3 Department of Oncology, Affiliated Hospital of Shaanxi University of Chinese Medicine, Xianyang 712000, China; 4 Shaanxi Provincial Clinical Research Center for Hepatic & Splenic Diseases, The Second Affiliated Hospital of Xi’an Jiaotong University, Xi’an 710004, China; 5 Key Laboratory of Environment and Genes Related to Diseases, Xi’an Jiaotong University, Ministry of Education of China, Xi’an 710061, China

**Keywords:** metabolic reprogramming, metabolic cancer, disulfidptosis, glucose starvation, natural products

## Abstract

Background: Metabolic cancers are defined by metabolic reprogramming. Although this reprograming drives rapid tumour growth and invasion, it also reveals specific metabolic vulnerabilities that can be therapeutically exploited in cancer therapy. A novel form of programmed cell death, known as disulfidptosis, was identified last year; tumour cells with high SLC7A11 expression undergo disulfidptosis when deprived of glucose. Natural products have attracted increasing attention and have shown potential to treat metabolic cancers through diverse mechanisms. Methods: We systematically searched electronic databases involving PubMed, Web of Science, Gooale Scholar. To ensue comprehensive exploration, keywords including metabolic reprogramming, metabolic cancer, disulfidptosis, natural products and some other words were employed. Results: In this review, we focus on the shared characteristics and metabolic vulnerabilities of metabolic cancers. Additionally, we discuss the molecular mechanisms underlying disulfidptosis and highlight key regulatory genes. Furthermore, we predict bioactive natural products that target disulfidptosis-related genes, offering new perspectives for anticancer strategies through the modulation of disulfidptosis. Conclusions: By summarizing current research progress, this review mainly analyzed the potential mechanisms of natural products in the treatment of metabolic cancer.

## 1. Introduction

Metabolic cancer is defined by the metabolic reprogramming within tumour cells, recognized as a critical hallmark of cancer development and maintenance [1,2,3]. Cancer cells, in contrast to normal cells, exhibit abnormal metabolic behaviours, such as excessive reliance on glycolysis despite sufficient oxygen availability, a phenomenon known as the Warburg effect [1,4]. Additionally, abnormalities in lipid metabolism and glutamine metabolism have been noted across various types of cancer [5]. These metabolic alterations not only support the rapid proliferation of tumour cells but also enhance tumour invasiveness and drug resistance by modulating the tumour microenvironment [6].

Despite the growing understanding of cancer metabolism, therapeutic strategies based on these insights remain significantly challenged. Currently, drugs targeting glycolysis and lipid metabolism are undergoing clinical trials [7]. However, due to the high metabolic heterogeneity of cancer cells, the efficacy of these drugs is often limited. Moreover, many metabolic targets also play essential roles in normal cells, resulting in substantial side effects that limit the broader application of these therapies [8]. The limitations of existing therapeutic approaches underscore the need for exploring novel targets and strategies. Recently, a research team led by Professor Boyi Gan at the MD Anderson Cancer Center identified and characterized a novel form of cell death, distinct from apoptosis, necroptosis, pyroptosis, ferroptosis, cuproptosis and autophagy-dependent cell death, termed “disulfidptosis” [9]. This discovery provides a new therapeutic avenue for cancer treatment. The study found that under glucose starvation, excessive consumption of reduced coenzyme II (NADPH) in tumour cells leads to abnormal accumulation of disulfides such as cystine, inducing disulfide stress, which triggers actin cytoskeleton contraction and cell death. Furthermore, cystine uptake is primarily mediated by the solute carrier family member SLC7A11 on the cell membrane. High expression of SLC7A11 significantly accelerates disulfidptosis in tumour cells under glucose starvation. This research suggests that targeting tumour cell glucose metabolism pathways could provide a basis for developing new cancer treatment models.

In recent years, natural products have attracted significant attention due to their di-verse biological activities and relatively low toxicity [10,11]. This review aims to explore the potentially bioactive natural products in targeting disulfidptosis. As the role of disulfidptosis in metabolic cancer becomes increasingly recognized, natural products may offer unique therapeutic advantages in targeting this emerging pathway. By summarizing current research progress, this review primarily examines the mechanisms through which natural products could treat metabolic cancer.

### 1.1. Characteristics of Metabolic Cancer

**Figure 1:**
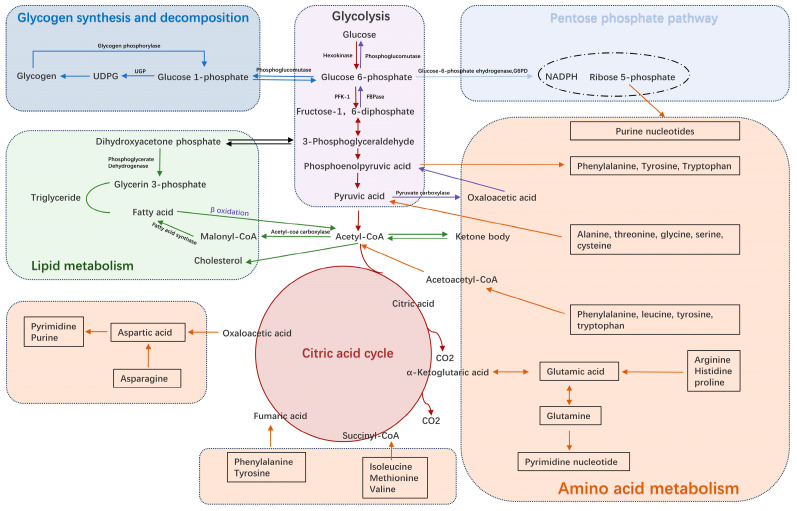
Metabolic pathways in normal cells. (UDPG: Uridine diphosphate glucose; UGP: Uridine diphosphate glucose -Pyrophosphorylase; PFK-1: Phosphofructokinase-1; FBPase: Fructose-1,6-bisphosphatase; NADPH: Nicotinamide Adenine Dinucleotide Phosphate Hydrogen; Co-A: Coenzyme A).

Unlike normal cells, metabolic tumours are characterized by metabolic reprogramming, which affects several key metabolic pathways to meet the demands of rapid tumour cell proliferation and division [5,8,12]. Figure 1 shows the main metabolic pathways including glucose, fat and amino acid metabolism in normal physiological states.

### 1.2. Glucose Metabolism Reprogramming

The abnormal glucose metabolism observed in tumour cells is called the “Warburg effect” [13,14]. This describes the preference of cancer cells for glycolysis over mitochondrial oxidative phosphorylation, even in the presence of oxygen. The Warburg effect not only provides sufficient energy for the rapid proliferation of tumour cells, but also provides intermediate products for the biosynthesis required for their growth, such as synthetic raw materials for amino acids and lipids. Anticancer treatments targeting glucose metabolism, such as those inhibiting key glycolytic enzymes, represent a major focus of current research [12].

### 1.3. Lipid Metabolism Reprogramming

As an important component of metabolic pathways, lipid metabolism in tumour cells has garnered growing attention. Compared with normal cells, tumour cells frequently display enhanced fatty acid synthesis and lipid storage capacity [15]. This not only supports the synthesis of cell membranes but also supplies essential precursors for signalling molecule synthesis. Abnormal lipid metabolism has also been reported to be closely associated with tumour aggressiveness and drug resistance [16,17]. Certain tumours, such as prostate cancer and renal cell carcinoma, are particularly dependent on alterations in lipid metabolism to sustain their growth. Studies have found that inhibiting lipid metabolism pathways can effectively suppress the progression of these tumours, offering new avenues for targeted therapy in metabolic tumours [12,18].

### 1.4. Amino Acid Metabolism Reprogramming

Amino acid metabolism is also significantly altered in metabolic cancers. Tumour cells exhibit an excessive dependence on specific amino acids, such as glutamine and arginine [19]. These amino acids not only provide an important source of energy but are also involved in many cell biological processes, such as resistance to oxidative stress and nucleic acid synthesis. As a major nitrogen source and energy supply, glutamine not only plays a key role in the nitrogen metabolism of tumour cells, but also promotes energy metabolism by converting it into α-ketoglutarate and entering the tricarboxylic acid cycle. Studies have found that inhibiting glutamine metabolism can significantly reduce the proliferation of tumour cells and induce their apoptosis [20]. Therefore, targeting amino acid metabolism has become an important anticancer strategy.

## 2. Metabolic Weaknesses

The metabolic reprogramming process acts as a “double-edged sword” in metabolic tumours. On the one hand, it serves as a pathway for energy acquisition to support tumour growth and survival. On the other hand, this reprogramming exposes a range of metabolic vulnerabilities, which form the theoretical foundation for targeted therapies against metabolic tumours.

### 2.1. Overdependence of Metabolic Pathways

Tumour cells exhibit an overreliance on glycolysis and specific amino acid metabolic pathways, positioning these as potential therapeutic targets. As one of the essential amino acids in the body, arginine is integral in protein synthesis in the body and is also involved in the synthesis of urea, ornithine, nitric oxide and other substances [21]. Many tumours, including MYC gene-driven small cell lung cancer, exhibit increased dependence on arginine [22]. When arginine is depleted, tumour cell viability is markedly reduced, making these cancer cells particularly sensitive to arginine deprivation therapies, such as arginine deiminase (ADI). Drugs targeting these key metabolic pathways can disrupt the energy supply and biosynthesis of cancer cells, thereby inhibiting tumour growth [23].

### 2.2. Accumulation of Metabolites

Metabolic reprogramming results in abnormal metabolite accumulation. When metabolic intermediates, such as lactate or lipid intermediates, accumulate, they can induce cytotoxic effects and alter the tumour microenvironment [24,25]. For example, some tumours accumulate intermediate metabolites such as α-ketoglutarate following the inactivation of mutated metabolic enzymes like iso-citrate dehydrogenase (IDH) [26]. These metabolites not only disrupt the intracellular metabolic balance but also inhibit the function of key enzymes, creating an immunosuppressive and pro-tumourigenic environment that promotes tumour progression. These metabolites can serve as biomarkers or therapeutic targets in cancer therapy. For instance, elevated lactate levels are associated with increased tumour aggressiveness and resistance to therapy, making lactate metabolism an attractive target for novel anticancer strategies [25,27]. Additionally, cancer cells often exhibit abnormal metabolic processes, including increased copper uptake. Studies have found that modulating copper homeostasis can selectively induce cancer cell death without affecting normal cells. For example, copper ionophores, such as dithiocarbamates, have shown potential in inducing copper-mediated cell death, and are particularly promising for cancers characterized by high copper accumulation, such as liver cancer [28]. In addition, copper can induce cell death via targeting lipoylated Tricarboxylic Acid cycle (TCA cycle) proteins, a new type of cell death regarded as “cuproptosis” that is distinguishable from established programmed cell death (e.g., apoptosis, pyroptosis, and ferroptosis) and provides novel insights into metabolic cancer therapy [29].

### 2.3. Abnormal Expression of Metabolic Enzymes

The expression of metabolic enzymes in tumour cells is frequently reprogrammed to meet their rapid proliferation demands [30]. As a key enzyme in the glycolysis pathway, normal cells primarily express pyruvate kinase M1 (PKM1), while pyruvate kinase M2 (PKM2) is highly expressed in tumour cells. PKM2 supports the glycolytic and biosynthetic requirements of cancer cells by favouring glycolysis over complete glucose oxidation for energy production [31]. By inhibiting the activity of these enzymes, the metabolic balance of tumour cells can be disturbed, thereby affecting their growth and survival [32,33].

As research on metabolic tumours advances, an increasing number of metabolic vulnerabilities are being identified and exploited for targeted therapies. Future anticancer strategies should not only inhibit the proliferation of tumour cells, but also disrupt their metabolic balance so that they cannot adapt to treatment pressure by reprogramming metabolic pathways. For instance, drugs that combine targeting glycolysis and glutamine metabolism are expected to show greater potential in treating refractory cancers [34]. By targeting the specific metabolic needs of tumour cells, we can effectively kill cancer cells without damaging normal cells, reduce the side effects of treatment, and improve patient survival rates.

## 3. Unique Cell Death in SLC7A11 High-Expression Cells

Professor Boyi Gan’s research team identified a novel type of cell death, which they termed “disulfidptosis”. This form of cell death is linked to the vulnerability of the cytoskeleton under disulfide bond stress. The research primarily investigates how cells with high SLC7A11 expression undergo this type of cell death during glucose starvation, driven by disulfide bond stress.

Initially, the researchers tested whether known cell death inhibitors could prevent the death of SLC7A11 high-expression UMRC6, H460, A549, and 786-O cells under glucose starvation. The findings showed that inhibitors of ferroptosis, such as ferrostatin-1 and deferoxamine, or apoptosis inhibitors, like Z-VAD-fmk, were not effective in stopping cell death. But inhibitors of disulfide bond stress, like dithiothreitol, β-mercaptoethanol, and T-CEP, were able to prevent cell death completely. This means that in cells with high SLC7A11 expression, the cell death caused by glucose starvation is closely tied to disulfide bond stress.

Further research showed that SLC7A11 high-expression cells quickly trigger a kind of cell death during glucose starvation that is different from apoptosis and ferroptosis. This death does not involve adenosine triphosphate (ATP ) loss or the formation of cysteine crystals. The researchers named this new type of cell death “disulfidptosis”.

Using a technique called stable isotope labelling by amino acids in cell culture (SILAC), the team analyzed how disulfide bond proteins change in SLC7A11 high-expression cells during glucose starvation. The results revealed that a large number of proteins formed disulfide bonds, especially proteins related to the cytoskeleton. Gene ontology analysis showed that proteins involved in the structure of the cytoskeleton and cell adhesion tend to form disulfide bonds when glucose is lacking. These proteins include FLNA/B, MYH9, TLN1, and ACTB, which have several cysteine residues in their peptides that form disulfide bonds.

**Figure 2:**
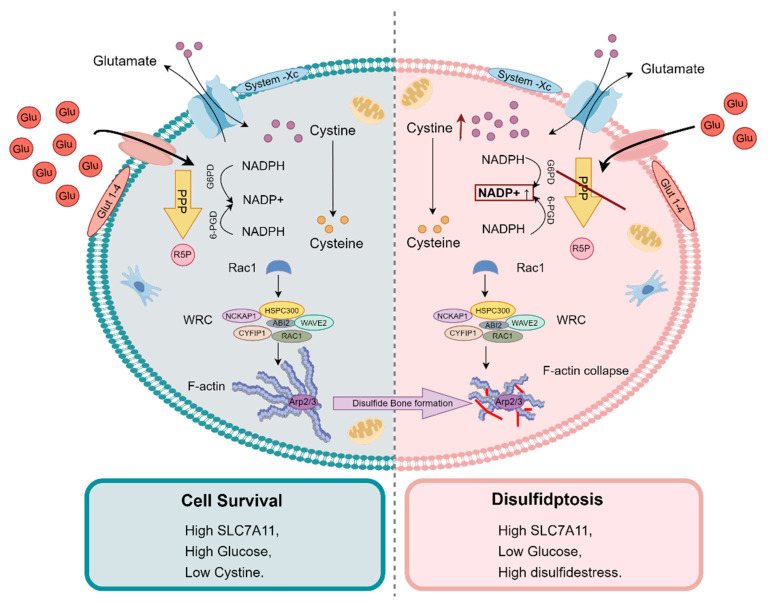
Molecular mechanisms of disulfidptosis. The study found that under glucose starvation, excessive consumption of reduced coenzyme II (NADPH) in tumour cells leads to abnormal accumulation of disulfides such as cystine, inducing disulfide stress, which triggers actin cytoskeleton contraction and cell death. Furthermore, cystine uptake is primarily mediated by the solute carrier family member SLC7A11 on the cell membrane. High expression of SLC7A11 significantly accelerates disulfidptosis in tumour cells under glucose starvation. (Glu: glucose; PPP: Pentose Phosphate Pathway; R5P: Ribose-5-phosphate; WRC: WAVE Regulatory Complex) (Figure created using Figdraw).

The study demonstrated that F-actin cytoskeleton disintegration occurs in SLC7A11 high-expression cells under glucose starvation, accompanied by significant disulfide bond formation. This disulfide bond stress affects the function and stability of cytoskeletal proteins, leading to cell death. Functional studies further revealed that inhibiting the WAVE regulatory complex (WRC), which promotes cytoskeleton polymerization, significantly reduces disulfidptosis. Conversely, activating the Rac signalling pathway promotes this form of cell death. Figure 2 illustrates the detailed molecular mechanism of disulfidptosis.

The study also showed that glucose transport inhibitors could induce disulfidptosis in SLC7A11 high-expression cancer cells, thus suppressing tumour growth. This finding provides a new potential strategy for cancer therapy, suggesting that targeting disulfidptosis in SLC7A11 high-expression cells could be valuable in future clinical applications.

By studying cancer cells with high SLC7A11 expression under glucose starvation using whole-genome CRISPR/Cas9 screening, the authors uncovered the vulnerability of the cytoskeleton to disulfide bond stress, highlighting the critical role of NADPH in maintaining the intracellular reductive environment, and identifying key genes likely to function in disulfidptosis (Table 1). This discovery not only deepens our understanding of cellular stress responses and death mechanisms but also offers a new perspective on how cancer cells adapt to nutrient deprivation. The identification of disulfidptosis opens up new avenues for exploring potential targets in cancer therapy.

## 4. Application Prospect of Natural Products in Metabolic Therapy Targeting Disulfidptosis

Natural products are naturally occurring compounds primarily derived from plants, microorganisms, animals, and marine organisms. These compounds are renowned for their structural diversity and wide-ranging biological activities, making them a significant resource for drug development [10,11]. Over the past 20 years, research on natural products has advanced significantly, particularly in the areas of anticancer [36], anti-inflammatory [37], and antiviral therapies [38]. Recently, natural products have shown great promise in inducing programmed cell death pathways, especially ferroptosis. By modulating intracellular iron metabolism and lipid peroxidation, natural products can effectively trigger ferroptosis. For example, epigallocatechin gallate (EGCG), a compound found in green tea, can induce ferroptosis by elevating intracellular reactive oxygen species (ROS) levels and promoting lipid peroxidation [39]. Other compounds, such as curcumin [40] and quercetin [41], have been found to enhance lipid peroxidation and induce ferroptosis by inhibiting glutathione peroxidase 4 (GPX4). In addition to ferroptosis, compounds like resveratrol, curcumin, and quercetin exhibit pro-oxidative effects in the presence of copper. These compounds can amplify copper-mediated reactive oxygen species (ROS) generation, leading to DNA damage and ultimately causing cancer cell death. Although copper overload can be toxic, chelation therapy can be strategically employed for the treatment of cancers with hypercupremic states, striking a careful balance between therapeutic efficacy and toxicity [42]. The ability of these natural products to enhance ROS generation in the presence of copper presents a novel therapeutic approach, particularly for cancers characterized by elevated copper levels, thus improving treatment selectivity and effectiveness [43].

As potential drug candidates, natural products targeting ferroptosis and cuproptosis provide promising avenues for the treatment of metabolic cancers. This raises the prospect of targeting disulfidptosis for therapeutic intervention. Future research could focus on screening natural products that are effective against tumours sensitive to disulfidptosis or using them to overcome tumour resistance [44] to chemotherapy by inducing disulfidptosis. Although there are no current reports of natural products directly affecting disulfidptosis, a deeper understanding of its molecular mechanisms, combined with in-sights from natural product research, could help identify natural compounds with therapeutic potential (Table 2).

### 4.1. Amino Acid and Nutrients Transport

SLC7A11 and SLC3A2 form a heterodimer, and together constitute the cystine/glutamate antiporter system, transporting cystine into the cell in exchange for glutamate [59]. This transporter plays a critical role in maintaining intracellular cystine levels and facilitating the synthesis of glutathione, which is the cell’s primary antioxidant [60]. In cancer cells, the overexpression of SLC7A11 promotes cystine uptake, thereby increasing resistance to oxidative stress and ferroptosis [61]. However, under glucose-starved conditions, excessive cystine uptake can lead to intracellular disulfide accumulation, triggering disulfidptosis. By upregulating the expression of SLC7A11 and SLC3A2, the cystine burden inside the cell increases, thereby promoting disulfidptosis in metabolically stressed cancer cells. Glabridin [46] has been reported to directly upregulate SLC3A2/SLC7A11, suppressing ferroptosis in diabetic nephropathy rat models. Unlike direct modulation of SLC7A11, many studies aim to regulate its expression by targeting upstream pathways, with NRF2 being the most notable molecular target. Rehmannioside A [48], Kaempferol [49], and Lycium barbarum polysaccharides [50] have all been shown to activate the Nrf2/SLC7A11/GPX4 pathway, mitigating ferroptosis.

On the other hand, glucose transporters GLUT1 and GLUT3 facilitate glucose entry into cells [62]. Inhibiting GLUT1/GLUT3 induces glucose starvation, increasing NADPH consumption and promoting disulfide bond formation, which in turn leads to disulfidptosis in cells with high SLC7A11 expression. Both Genistein [63] and Epigallocatechol Gallate [39] have been reported to bind to GLUT, reducing its transport efficiency and limiting glucose uptake into cells.

### 4.2. Cytoskeleton and Signal Transduction

NCKAP1, CYFIP1, WAVE2, ABI2, and HSPC300 encode components of the WAVE regulatory complex (WRC) [64], which plays a critical role in actin polymerization and cytoskeletal rearrangement. RAC1, a small GTPase, efficiently activates WRC [65], driving the Arp2/3 complex to regulate the actin cytoskeleton, promote lamellipodia formation and participating in cell migration and morphology maintenance. In disulfidptosis, upregulation of WRC-related genes and RAC1 leads to actin polymerization and disulfide bond crosslinking within the actin cytoskeleton, which is a key event causing cytoskeletal collapse and cell death. Enhancing the expression of WRC-related proteins or persistently activating RAC1 could promote disulfidptosis in metabolic tumours. Diallyl disulfide [53] derived from garlic has been shown to inhibit tumour invasiveness in gastric cancer by downregulating the TGF-β1/Rac1 pathway. Whether Diallyl disulfide plays a role in disulfidptosis remains unknown.

### 4.3. ER Stress Response

RPN1 is a subunit of the 26S proteasome, located in the 19S regulatory particle (RP) cap of the proteasome. It is a component of the endoplasmic reticulum-associated degradation (ERAD) system, which is responsible for degrading misfolded or accumulated proteins within the endoplasmic reticulum [66]. Additionally, RPN1 is part of the oligosaccharyl transferase (OST) complex and is critical for the N-glycosylation of glycoproteins, affecting protein folding and post-translational modification [67]. When disulfide bonds accumulate excessively within the cell, RPN1 helps mitigate ER stress by regulating the degradation of misfolded proteins. Silencing or inhibiting RPN1 exacerbates disulfide bond and misfolded protein accumulation, thus promoting disulfidptosis. ATF4, a key transcription factor in the cellular stress response, is essential for regulating endoplasmic reticulum stress, oxidative stress, and amino acid metabolism [68]. ATF4 controls the expression of various antioxidant genes, such as glutathione reductase (GSR) and glutathione peroxidase (GSH-Px), and is involved in the metabolism of glutamine and cysteine, which enables cells to counteract oxidative stress. During disulfidptosis, ATF4 is upregulated, enhancing the cells’ antioxidant defences against disulfide-induced death. Continuous upregulation of ATF4 expression may exacerbate endoplasmic reticulum stress under glucose starvation, thereby promoting disulfidptosis. Kuwanon H [54] has been shown to induce ER stress, leading to apoptosis and autophagy via ATF4 upregulation, and may also play a role in disulfidptosis.

### 4.4. Mitochondrial Function and Energy Metabolism

NUBPL, NDUFA11, and NDUFS1 are involved in encoding mitochondrial respiratory chain complex I [69,70,71], which plays a crucial role in oxidative phosphorylation and ATP production. LRPPRC regulates mitochondrial gene expression and affects energy metabolism [72]. Downregulating LRPPRC can inhibit tumour growth, invasion, and metastasis. OXSM participates in mitochondrial fatty acid synthesis [73]. Defects in mitochondrial function can increase oxidative stress and lead to energy depletion. Under glucose deprivation conditions, inhibiting these genes can heighten the susceptibility of tumour cells to disulfidptosis. Agrimol B [55] can induce autophagy reduction by downregulating NDUFS1 in liver cancer cells, leading to speculation that it could similarly influence disulfidptosis.

### 4.5. Glucose Metabolism

GYS1 is one of the key enzymes involved in glycogen synthesis, regulating the balance between glycogen production and breakdown [74,75]. In disulfidptosis, GYS1 expression is downregulated due to glucose starvation and reduced intake. Silencing GYS1 can further heighten cell sensitivity to glucose deprivation by reducing glycogen accumulation, inhibiting the glycolytic pathway, and promoting disulfidptosis. G6PD, 6-PGD, TALDO1, and TKT are key players in the pentose phosphate pathway (PPP) [76], which is crucial for maintaining NADPH levels, regulating redox balance, and resisting oxidative stress. Inhibiting this pathway depletes NADPH, exacerbates abnormal disulfide bond accumulation, and promotes disulfidptosis. R001 [56,57,58] is an unnamed natural compound extracted from the Vernonia cinerea plant. It has been shown to inhibit G6PD and TrxR1 functions in triple-negative breast cancer, limiting GSH synthesis and promoting oxidative stress. The inhibitory effect of R001 on G6PD suggests its potential application in promoting disulfidptosis.

### 4.6. Cell Division

PRC1 regulates cell division, ensuring correct differentiation and function during development [77]. Abnormal cell division can result in chromosomal instability [78]. Based on PRC1’s genetic functions, it is speculated that PRC1 may not directly participate in disulfide-induced cell death but rather plays an indirect role. It is possible that PRC1 influences the cellular decision to enter an oxidative stress state or proceed directly to disulfide-induced death by acting on cell cycle check-points [79]. Further research is required to confirm PRC1’s role in disulfide-induced cell death.

## 5. Prospects and Challenges

As mentioned above, therapies targeting disulfidptosis show great potential for treating metabolic tumours, especially when combined with natural product interventions. However, the development and application of natural products often face numerous challenges, with low bioavailability being one of the primary limitations for some of these compounds [80,81,82], which significantly hinders their clinical application. Many natural products, such as curcumin [83], have relatively low solubility due to their hydrophobic structures, limiting their absorption and bioavailability in the body. Additionally, some structurally complex natural products administered orally are poorly absorbed through the gastrointestinal tract, with only a small portion reaching systemic circulation [84]. Even if absorbed into the bloodstream, some natural products are often rapidly metabolized or eliminated due to enzymatic activity, limiting their ability to maintain effective concentrations in the blood [85,86]; their short half-life further limits their therapeutic potential.

Nanotechnology offers an effective strategy to overcome the challenges in drug delivery [80,81]. Using nanotechnology to encapsulate natural products can significantly improve their water solubility, stability and bioavailability. For example, nano-modification of curcumin is an effective strategy to address its low solubility, greatly enhancing its bioavailability and targeted delivery efficacy. Delivering resveratrol through biomimetic nano-systems not only improves its efficacy in treating colorectal cancer, but also addresses the challenge of low bioavailability of natural products [87]. In addition, solid lipid nanoparticles (SLNs), as a new type of nano-drug delivery system, can effectively extend the half-life of drugs in vivo and significantly enhance oral bioavailability [84]. SLNs significantly improve the solubility and bioavailability of dihydroartemisinin (DHA), greatly improving its anticancer effect in vivo [88]. Although the current nano-drug delivery system has shown great potential in the laboratory stage, it still faces many challenges in clinical application, especially in terms of biocompatibility, toxicity evaluation, and controlled drug release [89].

As a newly proposed form of programmed cell death, disulfidptosis currently has limited experimental research. Although studies have revealed some mechanisms underlying disulfidptosis, including the association between intracellular disulfide overaccumulation and redox imbalance, our understanding of its key molecules and regulatory pathways remains incomplete [3,9,35,90,91,92,93,94]. For instance, tumour cells originating from different primary tissues may exhibit variations in their sensitivity to disulfidptosis due to differences in tissue-specific functions and energy metabolism, such as glucose and lipid metabolism. Current research has primarily focused on cell types with high SLC7A11 expression, raising the question of whether disulfidptosis occurs in cells with low SLC7A11 expression. And if so, how does it differ from the characteristics of those in high-expressing cells? Moreover, while most studies focus on disulfidptosis as a distinct programmed cell death pathway, potential links between disulfidptosis and ferroptosis, which also involves SLC7A11, remain to be explored. The possibility of interactions or synergistic effects between disulfidptosis and other forms of regulated cell death, such as apoptosis and autophagy, also warrants further investigation. Additionally, the regulatory influence of the tumour microenvironment on disulfidptosis remains poorly understood. Developing in vivo strategies to induce glucose deprivation that can selectively target tumour cells while sparing normal tissues requires further research and optimization. Future studies should focus on elucidating these mechanisms and developing strategies for the precise targeting of tumour cells, and clinical trials based on disulfidptosis regulation are also necessary for targeting treatment.

In summary, although targeting disulfidptosis with natural products shows promise for the treatment of metabolic tumours, challenges remain in optimizing bioavailability, understanding the underlying mechanisms, and achieving clinical translation. As research into the molecular mechanisms of disulfidptosis progresses and the development of new drugs advances, this strategy could offer novel and more effective therapeutic approaches for precision oncology.

## Figures and Tables

**Table 1:** Disulfidptosis-related genes and functions [35].

Category	Gene	Gene Function	Expression in Disulfidptosis
Amino Acid/Nutrient Transport	SLC7A11	Responsible for amino acid (e.g., cystine) transport, involved in antioxidant stress response	Upregulated
	SLC3A2	Forms a heterodimer with SLC7A11, cooperatively transports amino acids	Upregulated
	GLUT1	Primarily responsible for transmembrane glucose transport, major carrier for glucose uptake	Downregulated
	GLUT3	High glucose affinity, primarily expressed in neurons, involved in glucose uptake	Downregulated
Cytoskeleton and Signal Transduction	NCKAP1	A member of the WAVE complex, which helps with changing the cell’s structure and is linked to cell movement	Upregulated
	CYFIP1	A member of the WAVE complex, which helps with changing the cell’s structure and is linked to cell movement	Upregulated
	WAVE2	A member of the WAVE complex, which helps with changing the cell’s structure and is linked to cell movement	Upregulated
	ABI2	A member of the WAVE complex, which helps with changing the cell’s structure and is linked to cell movement	Upregulated
	HSPC300	A member of the WAVE complex, which helps with changing the cell’s structure and is linked to cell movement	Upregulated
	RAC1	Small GTPase, regulates the cytoskeleton, plays a key role in cell migration and adhesion	Upregulated
Endoplasmic reticulum stress response	RPN1	Involved in post-translational modification and degradation of proteins, key protein in ER glycosylation	Upregulated
	ATF4	Transcription factor, plays a role in cellular stress response, regulates gene expression	Upregulated
Mitochondrial Function and Energy Metabolism	NUBPL	Mitochondrial assembly factor that is involved in putting together mitochondrial respiratory chain complex I	Downregulated
	NDUFA11	A part of mitochondrial respiratory chain complex I, which is involved in moving electrons and making ATP.	Downregulated
	LRPPRC	Regulates mitochondrial gene expression, influences oxidative phosphorylation process	Downregulated
	OXSM	Involved in fatty acid oxidation, part of the mitochondrial β-oxidation pathway	Downregulated
	NDUFS1	A main part of mitochondrial respiratory chain complex I, which is involved in oxidative phosphorylation	Downregulated
Glucose Metabolism	GYS1	Glycogen synthase, involved in glycogen synthesis, affects energy storage	Downregulated
	G6PD	Responsible for the first step of the pentose phosphate pathway, produces NADPH for reductive biosynthesis	Downregulated
	6-PGD	Involved in the pentose phosphate pathway	Downregulated
	TALDO1	Involved in the pentose phosphate pathway, generates nucleotides and NADPH	Downregulated
	TKT	Key enzyme in the pentose phosphate pathway, involved in carbon skeleton rearrangement	Downregulated
Cell Division	PRC1	Regulates the cell division process, particularly playing a role in cytokinesis during the final stages of cell division	Downregulated

**Table 2:** Potentially bioactive natural products that may target disulfidptosis.

Natural Product	Gene	Structure	Origin	Function	Reference
Curculigoside	SLC7A11	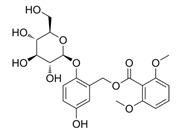	*C. orchioide*	Downregulating the SLC7A11/GPX4 signalling pathway promotes ferroptosis and improves Alzheimer’s disease	[45]
Glabridin	SLC3A2/SLC7A11	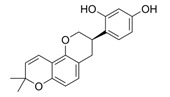	Licorice	Enhancing SLC3A2/SLC7A11 expression in diabetic nephropathy rats inhibits ferroptosis	[46]
Ginsenoside Rh2	IRF1	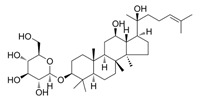	Ginseng	Upregulating IRF1 expression inhibits SLC7A11, enhances ferroptosis, reduces liver inflammation, and suppresses liver fibrosis	[47]
Rehmannioside A	NRF2	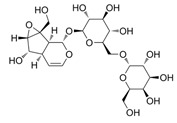	Rehmannia glutinosa Libosch	Activating the Nrf2 and SLC7A11/GPX4 signalling pathways inhibits ferroptosis and improves cognitive dysfunction after cerebral ischemia	[48]
Kaempferol	NRF2	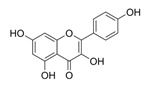	Green tea, Broccoli, Delphinium	Activating the Nrf2 and SLC7A11/GPX4 signalling pathways inhibits ferroptosis in OGD/R-treated neurons	[49]
Lycium barbarum polysaccharide	NRF2	Consisted of several monosaccharides (galactose, rhamnose, arabinose, glucose, mannose, xylose) and proteins	*L. barbarum*	Activating NRF2 promotes the expression of downstream targets such as HO-1 and SLC7A11, reducing ferroptosis	[50]
Genistein	GLUT1	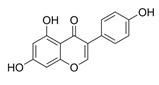	Soya	Binds to GLUT1 and inhibits GLUT1 transport	[51]
Epigallocatechol Gallate	GLUT1, GLUT3	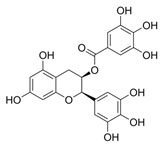	Green tea	Binds to GLUT1/3 and inhibits GLUT1/3 transport efficiency	[52]
Diallyl disulfide	RAC1	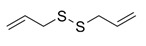	Garlic	Downregulates the TGF-β1/Rac1 pathway in gastric cancer, inhibits tumour invasiveness	[53]
Kuwanon H	ATF4	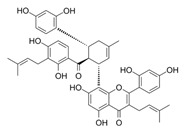	*Morus alba* L.	Upregulates ATF4, induces endoplasmic reticulum stress, leading to apoptosis and autophagy	[54]
Agrimol B	NDUFS1	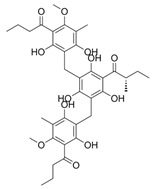	*Agrimonia pilosa* Ledeb	Downregulates NDUFS1, and increases mROS, inducing autophagy arrest	[55]
R001	G6PD	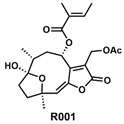	Vernonia cinerea plant	Inhibits G6PD and TrxR1 functions, leading to limited GSH synthesis and promoting oxidative stress	[56,57,58]

## Data Availability

Not applicable.
